# A laser pointer driven microheater for precise local heating and conditional gene regulation in vivo. Microheater driven gene regulation in zebrafish

**DOI:** 10.1186/1471-213X-9-73

**Published:** 2009-12-30

**Authors:** Mike Placinta, Meng-Chieh Shen, Marc Achermann, Rolf O Karlstrom

**Affiliations:** 1 Department of Biology, University of Massachusetts, Amherst, MA 01003, USA; 2 Department of Physics, University of Massachusetts, Amherst, MA 01003, USA

## Abstract

**Background:**

Tissue heating has been employed to study a variety of biological processes, including the study of genes that control embryonic development. Conditional regulation of gene expression is a particularly powerful approach for understanding gene function. One popular method for mis-expressing a gene of interest employs heat-inducible heat shock protein (hsp) promoters. Global heat shock of hsp-promoter-containing transgenic animals induces gene expression throughout all tissues, but does not allow for spatial control. Local heating allows for spatial control of hsp-promoter-driven transgenes, but methods for local heating are cumbersome and variably effective.

**Results:**

We describe a simple, highly controllable, and versatile apparatus for heating biological tissue and other materials on the micron-scale. This microheater employs micron-scale fiber optics and uses an inexpensive laser-pointer as a power source. Optical fibers can be pulled on a standard electrode puller to produce tips of varying sizes that can then be used to reliably heat 20-100 μm targets. We demonstrate precise spatiotemporal control of *hsp70l:GFP *transgene expression in a variety of tissue types in zebrafish embryos and larvae. We also show how this system can be employed as part of a new method for lineage tracing that would greatly facilitate the study of organogenesis and tissue regulation at any time in the life cycle.

**Conclusion:**

This versatile and simple local heater has broad utility for the study of gene function and for lineage tracing. This system could be used to control hsp-driven gene expression in any organism simply by bringing the fiber optic tip in contact with the tissue of interest. Beyond these uses for the study of gene function, this device has wide-ranging utility in materials science and could easily be adapted for therapeutic purposes in humans.

## Background

The study of gene function has been greatly facilitated by the ability to conditionally regulate gene expression at different locations and times throughout the life cycle. A variety of genetic tools that allow such temporal and/or spatial control of gene expression have been developed for study of gene function in several model organisms. These include site-specific recombination using cre or flp recombinases [1-3], tetracycline inducible systems [4], and the Gal4/UAS system [5,6]. Another system for conditional gene regulation takes advantage of the cellular heat shock response, which leads to the transcription of genes that allow cells to tolerate brief periods of stress, or to activate cell death pathways when these stresses are too extreme [7,8]. One particularly well studied heat shock inducible gene is the *hsp70l *gene, (formerly known as *hsp70*) which encodes a chaperonin that functions in protein folding mechanisms [9]. The *hsp70l *promoter activates transcription when cellular temperatures are raised by 10-15°C and has been used to analyze gene function at different times in the life cycle [e.g. [10-13]]. Global heat shock provides temporal control of gene expression [14], but does not allow the spatial control necessary for the analysis of region, tissue, and cell specific gene function. Methods for local tissue heating would allow the study of gene function in different tissues throughout the life cycle.

Laser-based techniques for controlled heating in small regions of biological samples are desirable, mainly because of the possibility for precise targeting and control of laser light. The most straight-forward approach uses existing microscopy setups to tightly focus laser light onto tissue to induce local heating [10,15-17]. The main drawback of this technique is the low absorption coefficient of biological tissues in the near-infrared wavelength range (close to that of water at ~1 cm^-1^), where typical solid state or diode lasers operate. For example, within 10 μm penetration depth only 0.1% of the laser power is deposited. To achieve temperature differences of 10-15°C, a typical laser with power in the Watt range is required, and this comes at a significant cost. In addition, precise control and calibration of the temperature is difficult, since exact laser light absorption of biological materials is not well known and random light scattering in biological samples affects the laser power at the target location. A solution for achieving heating with lower laser powers has been proposed by Zondervan et al., who used a metallic substrate that strongly absorbs laser light to heat the environment [18]. However, this approach is not very practical for biological studies as it does not allow three-dimensional positioning of the local heat source. Recently, an infrared laser was used to locally activate *hsp70 *transgene expression in *C. elegans *[19]. This method is effective, but has similar drawbacks, including difficulties in calibrating heating temperatures and high cost.

The zebrafish has emerged as a powerful genetic system for the study of vertebrate development, with the accessible embryo possessing many features that allow real-time observations and experimental manipulations. The zebrafish *hsp70l *promoter has been used to temporally regulate transgene expression by raising the temperature to 37°C at any time during development [14]. Both spatial and temporal regulation of a *hsp70l:GFP *transgene was demonstrated using a pulsed blue dye laser focused through a microscope lens [15]. However, this system has had limited utility due to issues of efficacy and cell viability. Spatio-temporal control of *hsp70l *transgene expression was also achieved using a simple soldering iron heating device in zebrafish [20], but spatial control is limited by the size of the soldering iron tip and this system is most useful for gene activation in superficial cells. In addition, the high thermal mass of this device necessitates general tissue cooling using a reservoir.

Our aim was to develop a simple micron-scale optical heater that would allow reproducible and convenient local heating anywhere in the zebrafish embryo without causing cell death. By combining an optical fiber based approach with a low-power laser source, we created an inexpensive local heat source that has a well-defined temperature and can be precisely positioned in biological tissue. Moreover, the area of local heating can be controlled by "pulling" optical fibers with a standard electrode puller, and these fine tips can then be positioned almost anywhere in the embryo or larvae. The temperature at the fiber tip can be precisely measured by a thermocouple-based thermometer. This microheater is simple, highly controllable, and induces gene expression without causing tissue damage or cell death. The ability to precisely control heating on the micrometer scale will have broad applications for the study of gene function and may have wide-ranging utility in the fields of medicine and materials science.

## Results and Discussion

### A simple optical fiber microheater

**Figure 1:**
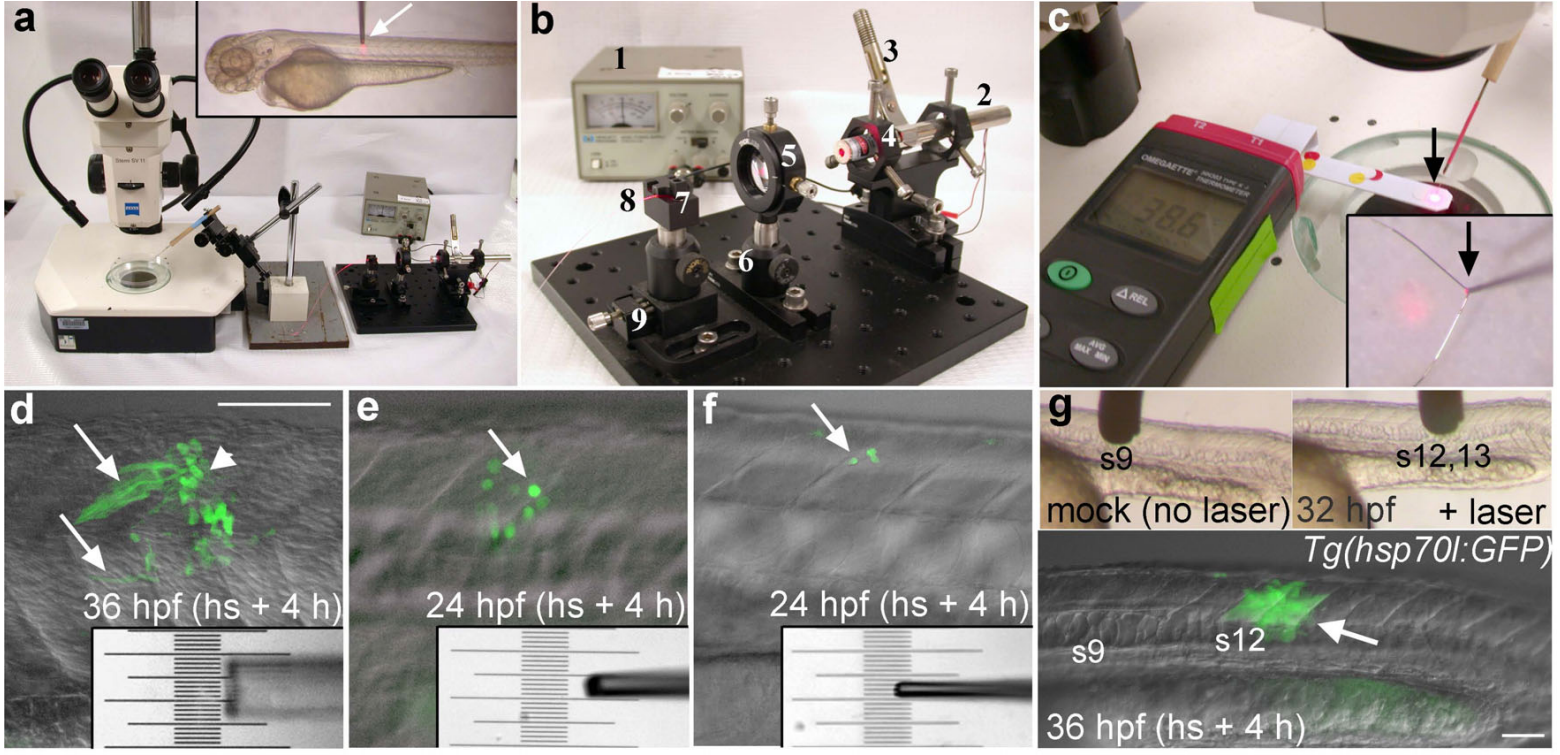
**The laser-pointer optical microheater and local *hsp70l:GFP *transgene induction**. **(a) **Laser pointer light was focused through a lens onto the end of a 13" long optic fiber that was mounted on a micromanipulator under a stereomicroscope. Inset shows the coated end of the optical fiber (arrow) contacting the trunk of a 72 hpf zebrafish larva mounted in agarose. **(b) **Close-up view of the laser pointer microheater assembly. (1) Variable power supply (2) 75 mW red beam laser pointer, **(**3) clamp to depress the laser pointer switch, (4) laser pointer holder, (5) lens, (6) post holder, (7) fiber clamp, (8) 50 μm core optical fiber, (9) translation stage. **(c) **Digital thermometer used to determine the temperature of the laser tip (arrow). Inset shows close-up of the heater tip contacting a k-type thermocouple (black arrow). **(d-f) **Examples of local transgene activation in *Tg(hsp70l:GFP) *embryos, lateral views of the trunk (see A inset). Somitic tissue was heated for 25 minutes using different sized fiber tips calibrated to 41°C. Insets show different sized tips over a micrometer. **(d) **GFP expressing muscle fibers (arrows) in a 36 hpf embryo 4 hours after heat shock (120 μm diameter (50 μm core) optical fiber tip). **(e) **GFP expressing cells (arrow) in a 24 hpf embryo 4 hours after heat shock using a 50 μm diameter tip. **(f) **GFP expression in a few lateral cells of a 24 hpf embryo 4 hours after local heat shock using a 30 μm diameter tip. **(g) **Mock treatment (no laser light) in the region of somite 9 (left inset) did not activate gene expression. Local heat shock in the region of somite 12 (right inset) in the same embryo activated transgene expression in somites 12 and 13 (arrow). Scale bars: d-f, 100 μm; g, 50 μm.

Optical fibers were first demonstrated in the late 19^th ^century, and then mainly used for internal illumination in medical applications. In the late twentieth century optical fibers became ubiquitous as a medium for telecommunications. Micron-scale optical fibers are now commercially available, allowing delivery of laser light on a biologically relevant size-scale. In our application, a 50 μm core/125 μm cladding multimode fiber serves as a flexible support and controllable heat source when coupled to a low power laser, namely a 75 mW laser pointer (Fig. 1a, b). After removing the fiber jacket, the tip of the fiber is coated with black ink that absorbs the laser light and converts it into heat. To estimate the absorbed power we measured the optical power after the fiber tip without and with ink coating. Assuming that absorption effects are dominant and that the reflectivity of the bare and the coated fiber is similar, we found that ~70% of the laser light is absorbed by the coated fiber tip (calculated as the difference in measured power output of the fiber tip with and without the ink coating). Such high absorption allows our device to operate with significantly lower laser powers than laser heating devices that use the absorption of the biological sample.

To analyze the functionality of our optical microheater, we calibrated the temperature of the coated fiber using a thermocouple-based thermometer in a drop of water at room temperature (~25°C) (Fig. 1c). The power to the laser was adjusted using a variable current source. We determined that the temperature increased linearly with laser power over a large temperature range with an inverse slope of ~0.48 mW/K. Hence, a laser with a power output below 10 mW is sufficient to establish temperature differences that are relevant for biological studies (5-15°C). Empirically, driving the laser with a current of ~55 mA resulted in a temperature of ~40°C which was used for the following experiments (it turned out the internal efficiency of the laser was less than 10%). It is noteworthy that only 53% of the laser power is converted into heating power as a result of the 75% fiber coupling efficiency and the 70% absorption of the ink coating.

### Local gene activation in zebrafish

To test and optimize the local heater we used the *Tg(hsp70l:GFP*) transgenic zebrafish in which expression of the fluorescent protein GFP is controlled by the *hsp70l *promoter. The coated optical fiber was brought into contact with an anesthetized zebrafish embryo or larva that was mounted in a drop of 1% low melting temperature (LMT) agarose (Fig. 1a inset). The tip was left in contact with the specimen for 20-25 minutes. Embryos were left mounted to monitor GFP gene expression using a fluorescent dissecting microscope. GFP expression was detected 3-4 hours after local heat shock (Fig. 1d-f). Embryos were then freed from the agarose and incubated at 28.5°C in embryo medium until the desired stage.

To vary the area of gene activation, optical fibers of different sizes were made by pulling the fiber on an electrode puller. The tapered glass could then be broken to the desired size, from 20 to 120 μm in diameter (Fig. 1d-f). An un-pulled fiber tip activated gene expression in a trunk region corresponding approximately to its 125 μm diameter (Fig. 1d). Smaller tips activated gene expression in correspondingly smaller regions (Fig. 1e, f). Mock treatments, in which transgenic embryos were mounted in LMT agarose and touched with the end of the optical fiber for up to 1 hour with no laser light, did not activate transgene expression (Fig. 1g).

**Figure 2:**
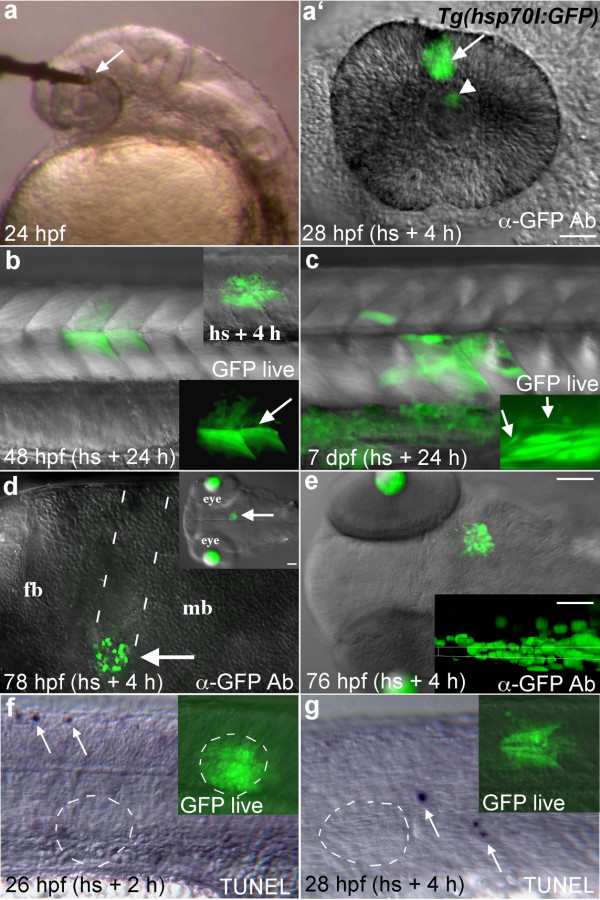
**GFP expression in *Tg(hsp70l:GFP) *embryos following local heat shock**. **(a) **A 50 μm diameter microheater tip (arrow) touching the eye of a 24 hpf embryo mounted in low melting temperature agarose. **(a') **GFP expressing cells (arrow) were seen in the heated region 4 hours later. GFP fluorescence is also seen reflected by the lens (arrowhead). **(b) **GFP expression (arrows) in a 48 hpf transgenic embryo heat shocked 24 h earlier in the trunk with a 100 μm optical fiber tip. Muscle fiber morphology in GFP expressing cells is normal (arrow in lower inset). Upper inset shows GFP expression 4 h after heat shock. **(c) **Transgene activation in a 7 dpf larva that was heat shocked at 6 dpf. Inset shows normal spinal neuron morphology (arrows) in a different individual. **(d) **A 50 μm tip was pushed into the brain of this embryo at 72 hpf to locally heat deep tissue (arrows, dotted lines show entry pathway). Lateral view of the brain, dorsal up. Transgene expression was activated with relatively little damage from the optical fiber. Inset shows the region of gene activation in a dorsal view. Lens tissue fluoresces in this transgenic line at these later developmental stages. **(e) **Dorsal view of GFP expression in the midbrain of a 76 hpf larva 4 hours after heat shock. Inset shows a resliced (xz) view of the GFP expression domain (maximum intensity projection along the y axis), dorsal up. GFP expression was activated in cells ~30 μm away from the dorsal surface that contacted the fiber optic tip. **(f, g) **TUNEL labeling in the trunk region 2 and 4 hours after heat shock, arrows indicate labeled apoptotic cells. No increased cell death was seen in the area of local heat shock (dashed circles). Insets show GFP expression just prior to fixation. Lateral views. Scale bars: a-d, f, g, 50 μm; e, 100 μm, inset, 30 μm.

To examine whether different tissues responded similarly to local heat shock, we heated regions of the head and trunk in *Tg(hsp70l:GFP) *embryos and larvae of different ages (Fig. 2). Transgene expression could be precisely activated in the eye, lateral hindbrain, midbrain, and trunk of embryonic or larval zebrafish (Fig. 2a-e). Over the course of these experiments the rate of transgene activation was ~85% (61/72 embryos heat shocked), through an age range from 10 hours to 7 days post fertilization. This rate approached 100% as we gained proficiency with the apparatus. Local heating in the trunk induced GFP expression in differentiated muscle fibers and differentiated neurons of the spinal cord, with cellular morphology suggesting these cells were healthy and undamaged by the heat shock (Fig. 2b, c). The GFP protein is quite stable, allowing labeled muscle fibers to be identified up to 6 days after heat shock (data not shown). Pulled fibers in the 10-50 μm range were small enough to allow for penetration into embryonic tissue, making it possible to activate gene expression in deep regions of the brain (Fig. 2d). Penetrating with the glass fiber caused remarkably little damage to brain tissue, and no transgene activation was seen along the path of entry.

To calculate the theoretical depth of gene expression we used a simple model of a one dimensional steady state heat flow based on Fourier's law of heat conduction: , in which *P *is the thermal power, *A *is the heated area, *k *is the thermal conductivity, and Δ*T *is the temperature gradient over the distance (depth) *L*. We approximated zebrafish tissue as water (*k*_*water *_= 0.6 Wm^-1^K^-1^) and assumed the heated area to be the total fiber tip surface including core and cladding. From the measured temperature of the ink coating as a function of absorbed laser power, we determine *P*/Δ*T *= 0.26 mW/K. Based on the different thermal conductivities of the fiber (*k*_*glass *_= 1 Wm^-1^K^-1^) and the zebrafish, we conclude that 38% of the heat flows into the fish and 62% into the optical fiber. Hence, we used *P*/Δ*T *= 0.098 mW/K to obtain the distance *L *= 75 μm over which the local temperature decreases to the global temperature of the fish (28.5°C). Knowing that *hsp70l *transgene expression is induced in zebrafish when *T *> ~37°C, the thickness of the expressing tissue is calculated to be 24 μm for a 41°C fiber tip. Our experiments show that the thickness of expressing tissue is between 20-30 μm (Fig. 2e) under these conditions, in good agreement with our calculations. The thickness of expressing cells could be increased to ~35-60 μm by increasing the tip temperature (data not shown), consistent with the model.

To verify that local heating did not induce cell death, we labeled apoptotic cells in embryos 2 and 4 hours after heat shock. No increase was seen in TUNEL labeled apoptotic cells in regions contacted by the local heater (Fig. 2f, g). Similar to un-manipulated embryos [21], only scattered apoptotic cells were labeled in the trunk region (arrows) and none of these happened to be in the area of local heat shock. If present, any dying cells must have been quickly eliminated and did not affect adjacent GFP expressing cells, in this case muscle fibers in the trunk (Fig. 2f, g insets). Increased cell death was seen with tip temperatures higher than 42°C (25 minute heating) or with heating times greater than 30 minutes at tip temperatures between 38°C and 41°C (data not shown).

### Local *hsp70l:GFP *transgene activation as a method for lineage tracing

**Figure 3:**
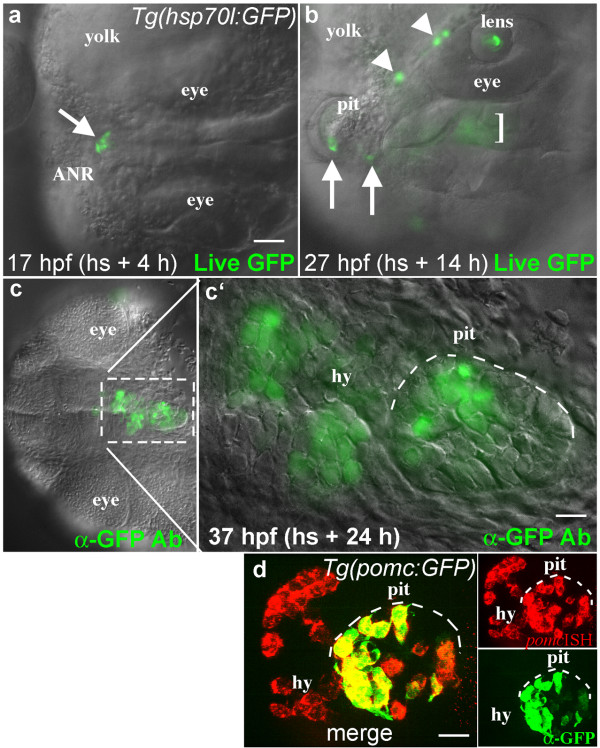
**Lineage tracing by local activation of the *hsp70l:GFP *transgene**. **(a, b) **Dorsal views of a live *Tg(hsp70l:GFP) *embryo, anterior to the left. **(a) **GFP expression in 4-6 cells at the anterior neural ridge (ANR, arrow) 4 h after local heat shock. **(b) **In the same embryo 14 h after heat shock, GFP expressing cells were present in known derivatives of this region including the forming pituitary placode (pit, arrows), marginal epidermis (arrowheads), lens, and hypothalamus (bracket, these ventral cells are out of focus in this dorsal view). **(c, c') **Ventral views (anterior left) of the same embryo following anti-GFP antibody labeling. 24 h after local heat shock, GFP positive cells were seen in the hypothalamus (hy) and adenohypophysis of the pituitary (pit, white dashed line). **(d) **In a different experiment, anti-GFP antibody labeling (green) was combined with fluorescent in situ hybridization to visualize gene expression (red). The endogenous *pomc *gene is expressed in endocrine cells of the hypothalamus (hy) and pituitary (pit). In the *Tg(pomc:GFP) *line, GFP is expressed only in POMC cells of the pituitary (pit), while POMC cells in the hypothalamus do not express GFP [29]. Merged image shows overlapping expression (yellow) in individual pituitary cells. Scale bars: a-c, 50 μm; c', 10 μm; d, 20 μm.

The stability of the GFP protein, combined with the ability to label small numbers of cells, led us to investigate whether the local activation of GFP with this device would provide an easy method of lineage tracing in older embryos and larvae. Other optically based lineage tracing methods such as those using caged fluorescein [22] or the photoconvertable Kaede protein [23] have considerable drawbacks, including background uncaging (caged flourescein) and the inability to perform subsequent labeling experiments to identify unique cell fates (Kaede). The GFP protein can be detected with antibody labeling and this labeling is preserved through standard in situ hybridization protocols (see Fig. 3d), allowing double or triple labeling strategies to define gene expression in GFP-labeled cells.

Our lab is interested in understanding early pituitary development in zebrafish [24,25]. Previous fate map studies have shown that the anterior lobe of the pituitary, the adenohypophysis, originates at the anterior margin of the CNS in both teleosts [26] and mammals [27]. As a proof of principle for local heat shock-based fate mapping, we heated a small region of the anterior margin at 13 hpf, activating GFP expression in 6-10 cells within 4 hours (Fig. 3a). 10 hours later, labeled cells were seen in the developing pituitary placode, as well as in the lens and neural tissue derived from the anterior margin (Fig. 3b). This is consistent with a previous fate mapping study in zebrafish showing that pre-placodal cells at the anterior midline contribute to both the lens and the adenohypophysis, with neighboring neural tissue forming the hypothalamus [28]. Another 10 hours later, this embryo was fixed and labeled using the anti-GFP antibody (Fig. 3c). Labeled cells were clearly seen in the lens, pituitary, and adjacent hypothalamus, as expected (Fig. 3c'). To determine the ultimate fates of GFP labeled cells in these lineage tracing experiments, it would be extremely useful to assay gene expression following cell differentiation. To determine whether it is possible to simultaneously detect gene expression and GFP labeling in individual cells, we took advantage of the *pomc:GFP *transgenic zebrafish line (*Tg(pomc*:*GFP*)) [29]. The endogenous *pomc *gene is expressed in differentiated POMC-secreting endocrine cells of the hypothalamus and pituitary gland [30] and can be visualized in the zebrafish using fluorescent in situ hybridization (Fig. 3d). In the *pomc:GFP *transgenic line, GFP is expressed only in differentiated POMC-secreting endocrine cells of the pituitary; hypothalamic POMC cells are not visualized because the hypothalamic-specific *pomc *enhancer element appears to be missing from the transgene [29]. Double fluorescent labeling in this line showed co-expression of GFP and the *pomc *gene in individual cell in the adenohypophysis (Fig. 3d), as expected. This demonstrates that fluorescent double labeling protocols will allow us to determine the fates of GFP expressing cells in lineage tracing experiments. This lineage tracing method can be adapted for any age and tissue, providing a simple method for fate mapping throughout embryonic and larval development.

## Conclusions

We describe a simple laser-pointer driven optical heater that allows reproducible local heating on the 20-100 micron scale. The optical microheater system is comprised of a low voltage power source connected to a laser pointer whose red beam is transmitted through a lens and optic fiber core and is absorbed by black ink to produce heat (Fig. 1). Local heating thus occurs at the tip of an optical fiber, whose temperature is controlled by adjusting laser power. Temperature at the tip can be precisely measured by a thermocouple-based thermometer. The area of gene activation correlates well with the size of the optical fiber tip (Fig. 1). This optical local heating system is easy to operate and provides efficient localized heat without general heating of the embryo.

Using zebrafish embryos and larvae we have shown that this new optical heater can reproducibly and safely heat living tissues without causing cell death. This device will have broad application for studies of gene function in living tissues, as it allows precise temporal and spatial regulation of heat-shock responsive transgenes without causing tissue damage or inducing apoptosis (Fig. 2). Local heat shock can be performed multiple times on the same embryo without causing global gene induction in the embryo (data not shown). The area of heating can be controlled by pulling optical fibers with a standard electrode puller, providing a great deal of flexibility in targeting gene activation domains. Precise targeting of gene activation, in combination with the longevity of the GFP protein, provides a simplified method for lineage tracing (Fig. 3) that would be particularly useful for the study of late stages of organogenesis, as well as the regulation of cell numbers and fates post-embryonically. Finally, it is simple to raise the tip temperature to a point at which cells are killed (data not shown), indicating this device could be used for local ablations. This microheater is simple, highly controllable, inexpensive, and induces gene expression without causing cell death.

Besides the demonstrated uses in embryology, this micrometer scale heater could have broad applications in research and human medicine. Micron-scale heating can also be used for material science applications to control or catalyze chemical reactions, allowing polymerization of plastics, or creating local melting conditions. Potential applications in human medicine include therapeutic tissue heating [31] and local activation of therapeutic genes [32] or compounds that could be done using well-developed arthroscopic procedures.

## Methods

### Microheater components

A 75 mW red beam laser pointer (Pulsar P75 Wicked laser) was connected to a variable power source (HP 6218C Power Supply 0-50V/0-.2A). Voltage was kept at 3V while the current was adjusted from 0-150 mA. The laser was attached to a mount (Astro-1) on a miniature breadboard (Thorlabs, *MB8 - Aluminum Breadboard, 8" × 8" × 1/2", 1/4-20 Threaded*). A focusing lens (Thorlabs LA1805) was placed in a translating mount (Thorlabs, LM1XY) approximately 2 cm in front of the laser pointer. The end of a ~13" long, 50 μm core optical fiber (Thorlabs AFS50/125Y 0.22-NA 50 μm Core Multimode Vis-IR Fiber) was placed in a fiber clamp (Thorlabs, HFF003) approximately 4 cm from the lens. The fiber clamp was mounted on a translation stage (Thorlabs, MS1) to adjust the distance between the fiber end and the lens. Power measurements were performed with a 13 PEM 001/J power meter (CVI Melles Griot).

As desired, optical fibers were pulled on Sutter Micro-Pipette Puller (Model P-97). Before pulling, the fiber optic coating was removed from a small region by brief flaming. The bare end of the fiber was coated in permanent black ink (Sharpie industrial permanent marker), taped to a wooden dowel, and mounted on a Narishigi Micromanipulator. Temperature at the fiber tip was adjusted by touching the tip to a k-type thermocouple (0.0005" diameter, Omega CHAL-0005) attached to a digital thermometer (Omegaette HH303 Type K J Thermometer). The thermocouple was mounted on plastic to prevent breakage and facilitate probe calibration. Current was adjusted to achieve a tip temperature of about 38-41°C (~55 mA depending on tip size). The temperature of the optic fiber tip was recorded before and after local heat shock.

### Zebrafish mounting and local heat shock

*Tg(hsp70l:GFP) *transgenic zebrafish lines [15] were maintained as previously described [33]. Transgenic embryos were grown in Embryo Raising Medium (ERM; 2M MgSO_4_, 2M KCl, 2M CaCl_2_, 5M NaH_2_PO_4_, 5M NaCl; [33]) at 28.5°C. For heat shock, dechorionated embryos or larvae were anesthetized using MS-222, embedded in a drop of 1% Low Melting Temperature (LMT) Agarose (Sigma) on a petri dish, and positioned using forceps before the agarose set. The coated tip of the fiber optic was brought into contact with the tissue and heated for 25 minutes. For deep tissue heating, 30-40 μm diameter fibers were pushed into embryonic tissue. As needed, skin over the heating site was disrupted using a sharp probe. In older embryos and larvae, a patch of skin was killed over the desired probe entry site by short exposure to a small drop of mineral oil (Sigma M8410) [34]. The skin healed readily during the recovery period. Embryos were kept mounted for 2-4 hours after heat shock and gene activation was documented on a fluorescent dissecting microscope. Embryos were freed from the agarose and incubated in ERM at 28.5°C. The fiber tip was recoated with ink after 7-10 uses, as needed.

### Antibody and TUNEL Labeling and Imaging

Embryos and larvae were fixed for 1 hour at room temperature in 4% paraformaldehyde and stored in methanol at -20°C. Immunohistochemistry was performed essentially as in [35] using a rabbit anti-GFP primary antibody (1:400, Invitrogen, A-11122) and a goat-anti-rabbit Alexa Fluor-488 (1:1000, Invitrogen, A-11008) secondary antibody. For double in situ/antibody labeling, anti-GFP antibody labeling was performed after fluorescent in situ labeling using the Fast Red fluorescent substrate (Roche Applied Science, 11496549001). For TUNEL labeling, embryos were fixed in 4% paraformaldehyde overnight and stored in methanol at -20°C. Labeling was performed following the manufacturers instructions (Roche applied science, 11684809910). Labeled embryos were cleared and mounted in 75% glycerol for DIC and fluorescent imaging using an Axioplan 2 compound microscope equipped with the apotome confocal system (Zeiss).

## Authors' contributions

MP built the local heat shock device and thermometer, optimized local heat shock conditions, and wrote the first drafts of the manuscript. MCS performed tunnel assays and in situ hybridization. ROK came up with the initial design of the local heater, helped optimize the procedure, and was the main author of the manuscript. MA contributed to the design and setup of the local heat shock device and modeled the heat flow. All work was done in the lab of ROK. All authors read and approved the final manuscript.
